# Can Manipulation of Durum Wheat Amylose Content Reduce the Glycaemic Index of Spaghetti?

**DOI:** 10.3390/foods9060693

**Published:** 2020-05-28

**Authors:** Mike Sissons, Francesco Sestili, Ermelinda Botticella, Stefania Masci, Domenico Lafiandra

**Affiliations:** 1 NSW Department of Primary Industries, Tamworth 2340, Australia; 2 Department of Agriculture and Forest Sciences, University of Tuscia, 01100 Viterbo, Italy; francescosestili@unitus.it (F.S.); e.botticella@unitus.it (E.B.); masci@unitus.it (S.M.)

**Keywords:** durum wheat, pasta, glycaemic index, high amylose, resistant starch

## Abstract

Resistant starch (RS) in foods has positive benefits for potentially alleviating lifestyle diseases. RS is correlated positively with starch amylose content. This study aimed to see what level of amylose in durum wheat is needed to lower pasta GI. The silencing of starch synthases IIa (SSIIa) and starch branching enzymes IIa (SBEIIa), key genes involved in starch biosynthesis, in durum wheat cultivar Svevo was performed and spaghetti was prepared and evaluated. The SSIIa and SBEIIa mutants have a 28% and 74% increase in amylose and a 2.8- and 35-fold increase in RS, respectively. Cooked pasta was softer, with higher cooking loss but lower stickiness compared to Svevo spaghetti, and with acceptable appearance and colour. In vitro starch digestion extent (area under the digestion curve) was decreased in both mutants, but much more in SBEIIa, while in vivo GI was only significantly reduced from 50 to 38 in SBEIIa. This is the first study of the glycaemic response of spaghetti prepared from SBEIIa and SSIIa durum wheat mutants. Overall pasta quality was acceptable in both mutants but the SBEIIa mutation provides a clear glycaemic benefit and would be much more appealing than wholemeal spaghetti. We suggest a minimum RS content in spaghetti of ~7% is needed to lower GI which corresponded to an amylose content of ~58%.

## 1. Introduction

Food products made from wheat are and continue to be a key source of human nutrition and pleasure to civilisations around the world. While bread products dominate, noodles and pasta, which are convenient, inexpensive, and easy to cook, continue to enjoy popularity across the globe. Pasta is prepared mostly from durum wheat semolina (*Triticum turgidum* subsp. *durum*) with a small rise in the consumption of wholegrain/wholemeal and bran containing pasta occurring over the last decade [1]. Regular pasta, which is made from semolina, is not an ideal source of dietary fibre as most has been removed during the milling of the grain. There is good evidence that regular consumption of wholegrain cereals offers a reduced risk of certain diseases like type 2 diabetes, cardiovascular disease, and certain cancers [2,3] and attempts are being made by the cereal industry to get this message out with mixed success across the world. Despite this knowledge, the daily intake of dietary fibre falls well short of daily recommendations, with more than 90% of the population of the USA, for example, not meeting target levels [4]. This is because the vast majority of wheat-based food is made from refined flour where the grain outer layers, which contain most of the fibre, have been removed in milling. However, such foods are preferred by consumers for their taste, sensory quality, and appearance, so refined wheat products, by default, constitute a major, although poor, source of fibre [5]. The key would be to modify the nutritional value of the refined wheat products, while largely retaining the same sensory qualities and consumer appeal. This is a better strategy than adding ingredients to improve fibre content like bran, wholegrain meal, legumes, gums, etc. In pasta, a meta-analysis of 66 studies where pasta was fortified to improve its nutritional value showed that enrichment levels up to only 10% can be tolerated before sensory properties are compromised, which will limit potential fibre improvement by this route [6].

Resistant starch (RS), which is that fraction of dietary fibre that escapes digestion and absorption in the upper gastrointestinal tract and flows to the large intestine, serving as a substrate for resident bacteria, has the potential to improve dietary fibre in foods. RS has been found to have many benefits in foods such as lowering glycaemic index, promoting satiety, prebiotic, hypocholesterolaemic, and more [7,8]. Several commercial sources of RS are available (Hi-maize, CrystaLean^®^, Novelose^®^, Amylomaize VII and others) to increase the dietary fibre (DF) of foods [8]. These have several advantages over conventional fibres being white, with bland flavour and of fine particle size, low calorie content with total dietary fibre (TDF) over 20%, absorb little water and can be more stable in food processing. Commercial sources of RS have been added to pasta and up to 10–20% can be incorporated, depending on the source, with some impact on sensory appeal while increasing fibre among other benefits [9,10,11,12]. However, an alternative to adding commercially synthesised RS is to elevate the natural levels of RS in the wheat, which are likely to be less expensive and more acceptable to consumers, being “natural” sources of high fibre.

As the increase of amylose in the grain has been associated with increased RS [13,14] and because of the association with reduced starch digestibility and increased fibre with many potential applications in food, the development of high amylose cereals was stimulated. In the last fifteen years, wheat scientists, targeting starch biosynthesis by plant genetics tools, have developed new lines in which amylose/amylopectin ratio results have been almost reversed compared to the normal starch [15,16,17,18,19,20]. In the durum wheat Svevo, the transgenic silencing of a single key enzyme (starch branching enzyme IIa-SBEIIa) increased amylose up to 75–80% vs. the 25–30% in the control [19]. SBEIIa is a transglycosylase enzyme that catalyzes the formation of α-1,6 branches within amylopectin. Hazard et al. [21] described a durum wheat line in which mutating the gene coding the same enzyme, resulted in a modest increase of amylose content in the starch. In one of the durum wheat lines, used in this work, the gene SBEIIa was knocked-down by a successful non-transgenic technology named TILLING [19]. TILLING is a reverse genetic approach that combines the use of mutagenesis (to increase the genetic variability) and molecular high throughput techniques (to identify mutations in target genes). The semolina of the new Svevo derived line (Svevo SBEIIa) reached 55% in amylose (on total starch) and resistant starch was found to be increased by up to 6.5%. Several authors have reported a significant increase in amylose consequent to the suppression of another enzyme, the starch synthase IIa (SSIIa), essential for the elongation of amylopectin chains [22,23]. In the SSIIa null wheats, the composition of the whole seed is altered [22]. Here, the modest increase in amylose is accompanied with a severe decrease in starch content and a large increase in total fiber. These characteristics open interesting possibilities for the production of new healthy foods [24].

The purpose of this work was to assess the pasta quality and glycaemic index of two novel, non-transgenic durum wheats with elevated levels of amylose in their endosperm with a view to showing the potential for reducing the glycaemic index of pasta while maintaining acceptable pasta technological properties.

## 2. Materials and Methods

### 2.1. Plant Materials

Durum wheat lines Svevo SSIIa and Svevo SBEIIa (referred to as SSIIa and SBEIIa) were previously produced [19,22]. The two lines along with the control (durum wheat cv. Svevo) were grown in open field at the Experimental Farm of the University of Tuscia, located in Viterbo, Italy (lat. 42°26′ N, long. 12°04′ E, altitude 310 m a.s.l.) in two different seasons: Svevo SSIIa along with Svevo in the season 2015–2016, whereas Svevo SBEIIa along with Svevo in the season 2016–2017. Nitrogen fertilization (180 kg ha^−1^) was split into three applications: the first was given before sowing as di-ammonium phosphate (20% of total N applied), the second when the first node was detectable above ground as urea (50% of total N), and the third 25 days later as ammonium nitrate (30% of total N). Weather data show a typical pattern at this location (Supplementary Table S1).

### 2.2. Sample Preparation and Analytical Methods

Wheat was cleaned, conditioned to a water content of about 16.5% and left to moisten overnight. Standard milling was performed in a Buhler MLU 202 mill (Buhler, Utzwil, Switzerland) with three breaking and three sizing passages [25]. Semolina protein was determined by Dumas combustion using a Leco TruMax CN combustion nitrogen analyser (Leco Corp. St. Joseph, MI, USA) calibrated with sulfamethazine [26]. Semolina moisture was determined by the approved Method 44–15A [25]. Swelling power was measured as described elsewhere [27] in duplicate. The amylose content of the semolina, and resistant starch and total starch content of ground pasta (coffee grinder, sieved across a 250 µm screen) were assayed in duplicate using Megazyme kits (Deltagen Australia, Melbourne, Australia). Starch gelatinization parameters (enthalpy, onset, peak and end temperature) were performed on isolated starch as described previously [28] except the temperature was ramped up to 120 °C at 10 °C/min. Flour water absorption, adjusted to 14% mb (FWA, 14% mb) was determined using a MicroDoughLAB (Perten Instruments, Australia) fitted with a 4-g bowl, mixing at 120 rpm to target peak 650 FU in duplicate [25]. The particle size distribution of the semolina was measured using a vibratory sieve shaker (Fritch, Analysette 3 sparatan, Germany) adjusting the amplitude to 2.0 mm with a run time of 3 min using screens of apertures 500, 425, 315, 250, and 180 µm and the amount retained on each screen was collected and weighed.

### 2.3. Pasta Preparation and Evaluation

Spaghetti was prepared as previously described [29] but with adjustment to water added to make the dough based on the water absorption of the semolina to account for the higher water absorption of high amylose flours [30]. For 1 kg of semolina, the amount of water added for Svevo was 290 mL; SSIIa 333 mL and SBEIIa 349 mL. Dough blends were prepared in a premixing chamber for 15 min then the dough transferred to a pasta extruder fitted with a 1.82 mm spaghetti die (Appar Laboratorio, Rome). Wet spaghetti was transferred to a drying cabinet (TEC 2604, Thermoline Scientific Equipment, Smithfield, Australia) and dried using drying program up to maximum 65 °C. Dried pasta was stored in sealed plastic bags at room temperature until required for analysis.

All pasta samples were cooked to their fully cooked time (FCT), the time taken for the central starch core to disappear [25] and assessed for texture (cooked firmness, overcooking tolerance (100 × [firmness at FCT-firmness at FCT plus 10 min overcooking/firmness at FCT], stickiness), cooking loss and water absorption as described previously [31]. For firmness and overcooking tolerance, 12 replicate tests were performed per sample, for stickiness, a minimum of four replicate analyses per sample, and for cooking loss and water absorption of pasta, duplicate analyses were collected. The colour of uncooked 7 cm spaghetti strands aligned to minimize air spaces enough to cover the Minolta Chroma meter CR-410 detector (Biolab Australia, Sydney) was performed with a minimum of 4 replicate readings calibrated with white tile supplied by manufacturer. Measurements were L* (brightness, 100 = white; 0 = black), a* (positive value is redness and negative value is greenness), and b* (positive value, yellowness; negative value, blueness). A commercial sample of wholemeal spaghetti was used for comparison (15% protein, 62% carbohydrate, 3.5% fat and 9% dietary fibre).

### 2.4. In Vitro Starch Digestion of Pasta

Starch digestion of the samples was determined based on previous work [32]. Six pasta strands (typically 35–50 mm in length) for each sample were cooked in 36 mL of RO water to their FCT then cooled in water and trimmed to ~5 mm length. About 9–12 pieces were added (to standardize digestions 90 mg of starch was subject to digestion) to three 100 mL conical flasks (2 replicates samples and one control—no enzymes added) to which 6 mL of pre-heated RO water was added and 5 mL of pepsin solution (1 mg/mL in 0.02 M HCl) except for control with 0.02 N HCL added. Flasks were incubated with shaking at 1400 rpm for 30 min in a water bath held at 37 °C. To terminate the reaction, 5 mL of 0.2 M sodium acetate buffer (pH 6.0) was added to each flask followed by addition of 5 mL α amylase/amyloglucosidase (AA/AMG) solution and 5 mL of buffer to controls then incubated for 360 min at 37 °C. During the incubation, at intervals, a 0.1 mL aliquot was removed from the reaction mixture and mixed with 0.9 mL of ethanol (to terminate the enzyme reaction). This mixture was assayed for glucose using the Megazyme GOPOD reagent kit as per instructions. Absorbance at 510 nm was recorded using a UV mini −1240 Spectrophotometer (Shimadzu). Glucose content (mg/mL) = corrected sample absorbance (test sample absorbance–control absorbance)/absorbance glucose standard.

The starch digested (%) was calculated as:

Glucose content × 10 × 21 × (162/180) × (100/90)

where 10 is the dilution factor (0.1 mL of reaction mix added to 0.9 mL ethanol), 21 the dilution factor (1 mL to 21 mL reaction mix), 162/180 the molecular weight ratio when converting from starch to glucose, 90 the quantity of starch present in reaction mix in mg, and 100 to convert to %.

### 2.5. In Vivo Glycaemic Index (GI) Testing

This measurement was contracted to SUGiRS laboratory (Sydney University glycaemic index research services, https://www.glycemicindex.com/testing_research.php). In brief, 10 healthy subjects, non-smoking, aged 19–46 years with an average body mass index of 21.5–22.1 kg/m^2^ within the healthy range, were selected for the study on two separate occasions (samples Svevo 2016 and SSIIa pasta tested in 2017 and Svevo 2017 and SBEIIa pasta tested in 2019). The reference food and two pasta samples were served to participants (who had fasted overnight) in fixed test portions containing 50 g of available carbohydrate. Pure glucose (Glucodin™ powder, Valeant Pharmaceuticals, NSW) dissolved in water was used as a reference food and was consumed by each participant on three separate occasions, but participants only consumed the test pasta on one occasion. Each test portion of pasta was prepared shortly before being required by weighing appropriate amount of dry pasta and cooking in boiling water to FCT, drained, rinsed under cold water and then served to a participant with a glass of 250 mL of water. Participants were required to consume all food and fluid served.

A fasting blood sample is collected and then the test or reference sample consumed after which additional blood samples are collected at regular intervals over the next 2-h period. The same procedure is repeated in the same group of people on another day after they have consumed a portion of the reference glucose. The glucose was consumed on the first, third and fifth test sessions and the pasta samples were consumed in random order in between. The night before each test session, participants ate a regular evening meal based on carbohydrate-rich food and then fasted for at least 10 h overnight. Blood samples collected were centrifuged for 45 s immediately after collection and the plasma layer removed and stored at −20 °C for later analysis. Plasma glucose was measured in duplicate using a glucose hexokinase assay and clinical chemistry analyser. A GI value for the test pasta is calculated by expressing the 2 h blood glucose area under the curve for the test pasta as a percentage of the area produced by the glucose (GI = 100).

### 2.6. Statistical Methods

Data were analysed using the statistical programme GenStat version 17.1.0.14713 with a generalised linear model and the means were tested for significant differences by the least significant difference statistic (LSD), *p* < 0.05. Data were checked for normality and a Pearson correlation analysis was performed to examine the relationships between measured parameters.

## 3. Results and Discussion

### 3.1. Impact of Elevated Amylose on Pasta Technological Properties

Given that the high amylose samples were developed and field trialled in different years, Svevo as control was grown in both the 2016 and 2017 seasons to be control for the SSIIa and SBEIIa, respectively. Typical amylose contents of durum wheat range from 26% to 32% [33] but this can vary with seasonal conditions with Svevo amylose in this range (Table 1). As previously demonstrated, the elimination of SSIIa and SBEIIa in durum wheat by mutations created in the *SSIIa* and *SBEIIa* genes results in seeds with a higher amylose content compared to the wild type ones [19,22,34] as we obtained (Table 1), with amylose in SSIIa increased by ~29% and in SBEIIa by ~72% compared to Svevo. Similar results were obtained in bread wheat [20,35]. The elimination of starch biosynthetic enzymes can cause a reduction in starch synthesis and reduced seed weight and the total starch in the pasta of the high amylose (HA) genotypes (SSIIa and SBEIIa) was decreased significantly (Table 2). The protein content of the Svevo semolina differed only slightly across the two growing seasons while HA genotypes showed higher protein content than their corresponding Svevo (Table 1) probably due to the starch reduction and smaller grains (data not shown). This would explain the higher grain protein content of the HA lines as observed in other HA material [36]. Botticella et al. [23] reported a smaller grain weight, 25–45% reduction in starch content and concomitant increase in protein in the genotype Svevo SSIIa. Hogg et al. [35] also reported higher grain protein, lower starch content and kernel weight, and lower swelling power in their SSIIa triple mutant, consistent with our results for Svevo SSIIa.

It is known that the ratio of amylose to amylopectin affects the water absorption of flour [30,37] and this needs to be determined to optimise the water addition for pasta preparation. Both HA semolina’s have a much higher water absorption than Svevo (Table 1) with SBEIIa being a few percent higher than SSIIa. A similar behaviour was observed with waxy wheat flour [30], that attributed the increase in water absorption to the higher dietary fiber content of HA starch. While total fibre was not measured in the SSIIa genotype, it was significantly higher in SBEIIa compared to Svevo (Table 1). However, the increase in the resistant starch content of the HA pasta was significantly higher than Svevo genotypes (Table 2) supporting the increased fibre content of the HA genotypes as RS is a fibre. The SBEIIa has a much higher RS content than SSIIa pasta with both having a 35- and 2.8-fold increase above Svevo, respectively. The semolina swelling power of both HA lines were lower than Svevo (Table 1). It is known that this parameter is negatively correlated with the amylose content because amylopectin is responsible for the starch swelling and amylose acts as a diluent [38,39]. These changes are expected to have an impact on pasta texture.

**Figure 1:**
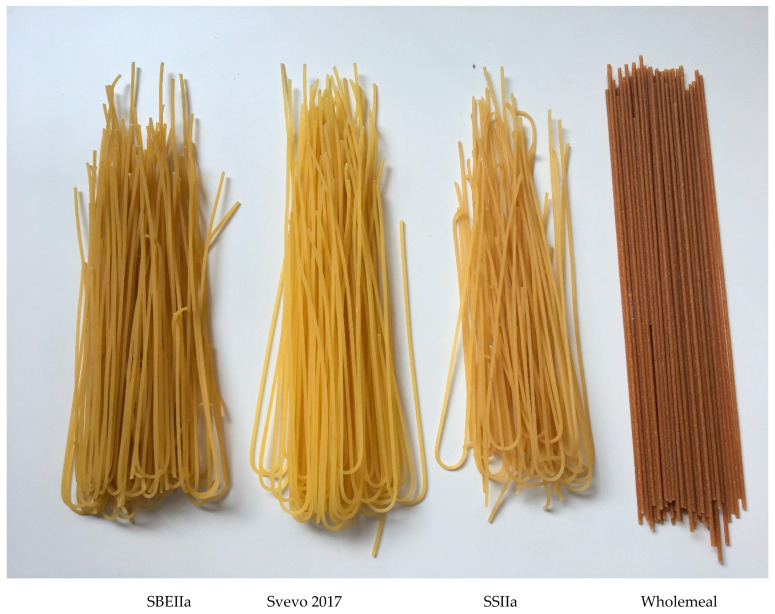
Spaghetti appearance of genotypes compared to commercial wholemeal pasta. From left to right SBEIIa, Svevo 2017, SSIIa, Commercial Wholemeal.

Pasta in the form of spaghetti, a common shape consumed worldwide, was evaluated for its colour, cooked texture, and cooking quality parameters, which are important to consumers of pasta. Pasta cooking quality can be assessed for features typical of good quality pasta which should be firm and resilient, with minimal cooking loss and surface stickiness and increase in volume after cooking to provide an acceptable mouthfeel. The yellowness of pasta is of aesthetic importance for consumer acceptance and marketing whereas redness and brownness are considered undesirable [40]. The appearance of the pasta (Figure 1) shows both HA pastas have a very similar appearance to the Svevo. SBEIIa was duller while SSIIa was closer to Svevo in visual colour but a little darker (duller). In contrast, a commercial wholemeal pasta example appears much darker, red and more grainy.

Analysis of the colour parameters of the dry pasta show the HA samples are a little duller (lower L*), with more redness (+ve a* values) and are less yellow than their Svevo pasta controls (Table 2) consistent with the visual appearance. There were subtle differences between Svevo in 2016 and 2017 seasons and this is due to environmental effects on colour of pasta or semolina [41]. The SBEIIa semolina was duller and appeared to be browner than Svevo and SSIIa. This could be related to the higher ash content, as noted for HA durum [24]. Indeed, we found higher ash contents in the HA semolina compared with Svevo, consistent with the higher amylose content correlated to lipid amount (Table 1). The duller HA pasta is likely due to their higher protein content since noodle brightness is negatively correlated to protein content [42] but also more bran flecks in the semolina. Clearly the HA pasta have a much more desirable appearance than the commercial wholemeal example we selected from local supermarket, which is much redder, duller, and less yellow. In agreement with our results, Hazard et al. [43] observed a deterioration in pasta color made from high-amylose semolina from SBEII mutants.

Pasta cooking time is affected by the shape, diameter and density of the strand and cooking method, which were controlled in this study. Cooking time is very much influenced by the rate of water migration into the strands and degree of starch gelatinisation, which can be affected by starch composition and starch swelling [39,44]. Both HA pasta had reduced FCT with SSIIa having the lowest (Table 3). This is most likely due the HA pasta having less starch to gelatinise, although this does not explain why SSIIa has shorter FCT than SBEIIa. It is likely the reduced starch swelling (lower swelling power, Table 1) caused by less amylopectin is responsible since it would allow more rapid gelatinisation (lower onset) and shorten cooking time.

Indeed, onset gelatinisation (T_onset_) of HA pasta was at a lower temperature for SBEIIa but ~6–7 °C lower for SSIIa (Table 4), while end set gelatinisation (T_end_) was much higher for the HA pasta’s especially SBEIIa. Lower gelatinisation enthalpy (ΔH) and greater gelatinisation range for high amylose mutant starches compared to the corresponding wild-type control in material with a wide range in amylose content (36–93%) has been reported [28] confirming our results. We found in the Svevo HA pasta no significant decrease in enthalpy, although it tended to decline but a very wide range in gelatinisation temperature compared to Svevo.

**Figure 2:**
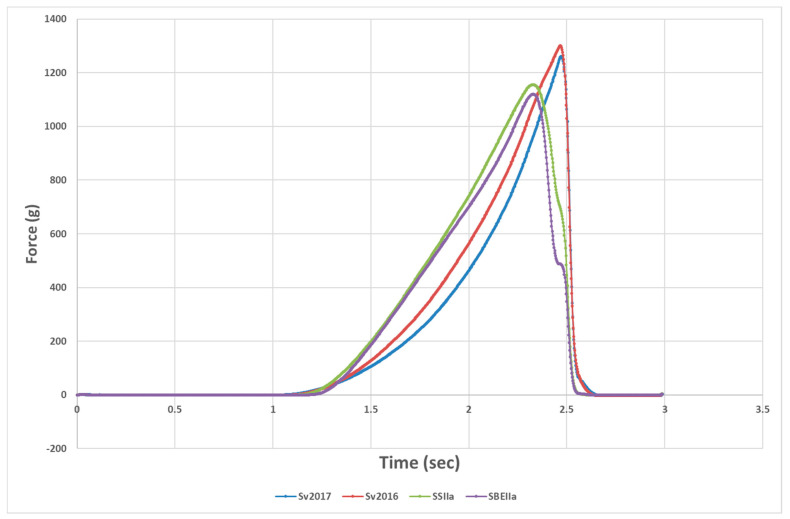
Typical profile for firmness measurement of HA and control genotypes.

The texture analyser compression test tries to evaluate the sensory equivalent of the “first bite” (compression of the strand by the incisors) and the force versus time curve provides two parameters, the height at the peak force and the work to cut or area under the peak (Figure 2). Pasta firmness peak height for the HA lines was reduced compared to Svevo while area under the compression curve was lower in SSIIa compared to its control, but not for the SBEIIa and its control (Table 3). Firmness is correlated to protein content [45,46] and one would expect a higher firmness in the HA pastas. However, adjustment for this shows that peak height/protein (PH/P) for the HA pastas was still significantly lower than the Svevo controls while both HA pasta had similar firmness with SBEIIa significantly softer. While the protein content of the two Svevo semolina samples was almost identical, pasta firmness was lower for Svevo 2017 as other factors come into play, affecting firmness beyond experimental error. The HA pastas have less amylopectin, which would reduce swelling, and this was noted in significantly lower pasta swelling (Table 1) or water absorption (Table 3). Reduced swelling and higher protein content would be expected to make the cooked firmness increase [47] but the opposite occurred. This was in contrast to what has been observed in other works, where an increased firmness was reported in pasta prepared with high amylose semolina [24,37,43,48]. However, Aravind et al. [9] added commercial RSII or RSIII to pasta with no clear effect on pasta firmness. Sozer et al. [44] added green banana with high RS to pasta with no effect on hardness. It is not easy to explain these differences, but sample differences and methods of assessing firmness could be explanations. It is also possible this type of measurement does not reveal the full impacts of high amylose starch in pasta and perhaps a better measure would be the elasticity of the pasta.

Typically consumers overcook pasta and this leads to a reduction in firmness as any ungelatinised starch in the central core becomes fully gelatinised and the pasta can swell more making it softer. The overcooking tolerance or resistance to firmness reduction is a good measure of tolerance and pasta should resist overcooking while still retaining al dente (having some firmness to the bite), and the lower this value, the more tolerant the pasta is to firmness loss due to overcooking, which is desirable. There were small but significant differences between the pasta with SBEIIa having the best tolerance, while the SSIIa pasta overcooking tolerance was not different to its Svevo control (Table 3). Hogg and colleagues [24] noted its high amylose durum (SSIIa null) was found to be more resistant to overcooking compared with the wild type while our Svevo SSIIa pasta had same tolerance to Svevo 2016.

There was a tendency for the HA pastas to have reduced stickiness with SSIIa pasta having lower area, but not significantly different peak height, although tending to be lower, compared to Svevo. While SBEIIa showed the opposite trend with a lower peak height, but not area compared to its Svevo control. Both HA pastas had equivalent stickiness. Soh et al. [37] using reconstitution studies found no change in pasta stickiness made from high amylose maize starch (27–74% amylose), whereas lowering the amylose content (from 23% to 0.7%) makes pasta stickier [49]. Aravind et al. [9] added commercial RSII and RSIII to pasta formulations and found no impact on pasta stickiness. Both HA pasta had significantly higher cooking loss than their Svevo controls with no significant difference between SSIIa and SBEIIa pasta (Table 3). However, the magnitude of the cooking loss is at an acceptable level (7–8%). A higher cooking loss was observed in SBEIIa nulls by Hazard et al. [43], but again they report around 6.4–6.7% results, similar to our own. The higher cooking loss could be related to the higher amylose content and its ability to leach out of the pasta during cooking [43], but this did not lead to increased pasta stickiness. Higher amylose in the pasta also affected water uptake in the pasta being significantly lower than the respective Svevo controls. This could be related to the reduced tendency for HA starch granules to swell as they contain less amylopectin and have tightly packed granules that are more resistant to swelling [50].

Overall, the HA pastas show slightly reduced firmness and increased cooking loss with inferior colour to Svevo but with reduced FCT and slightly lower stickiness. In conclusion, both highlight an acceptable quality. The colour may be an issue and require further breeding to improve but these HA pastas are superior to the commercial wholemeal pasta chosen for comparison. Nevertheless, the HA pasta improves RS and hence dietary fibre by 128% without the need for adding bran and would achieve more consumer acceptance being similar in appearance to regular, normal amylose content pasta. The nutritional value of pasta from both HA lines are improved in terms of their increase in resistant starch, which is a fibre, and lowering of the GI (for SBEIIa), see below. The total dietary fibre content of Svevo 2017 and SBEIIa were measured and showed significant increase with SBEIIa consisting of 67% insoluble and 33% soluble fibre (Table 1). In every 100 g dried pasta cooked, there is 5.7 g total dietary fibre which meets 23–19% of the recommended fibre daily requirement (2–30 g/d) (https://www.nutritionaustralia.org/national/resource/fibre). Further work is required using a trained sensory panel to determine the differences between the pastas and a consumer panel to determine the preferences for the four different pastas (standard, SSIIa, SBEIIa, wholemeal).

### 3.2. Impact of Elevated Amylose on Pasta In Vitro Starch Digestion

**Figure 3:**
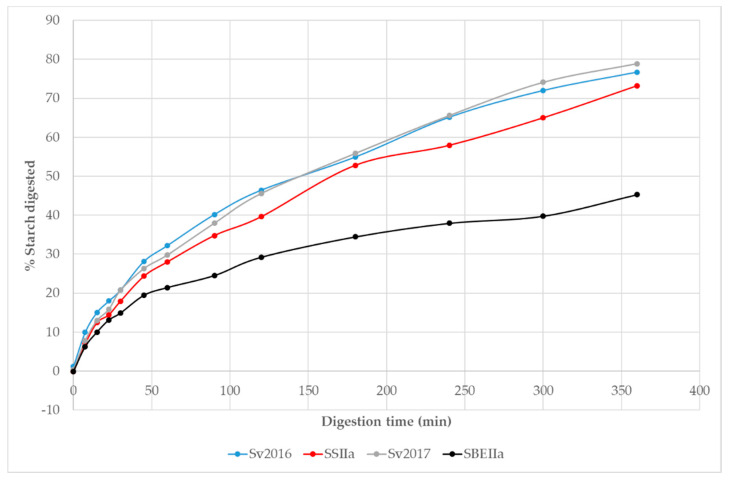
Digestibility curves obtained for cooked pasta of HA and control genotypes digested by α amylase/amyloglucosidase.

The HA pasta’s had elevated RS compared to Svevo, with SBEIIa achieving a 35 fold increase relative to Svevo 2017 while SSIIa achieved a 2.8-fold increase relative to Svevo 2016 (Table 2). Elevation of RS content in endosperm can be achieved by reducing SSIIa activity, enhanced GBSSI activity or hindered SBEII activity [51]. We were interested to know the starch digestion kinetics of the HA pasta samples that allows to quantify rate and extent of digestion which can be achieved using an in vitro method. Based on other studies [52,53], we expect a reduction in the extent of starch digestion in the HA pastas compared to Svevo. Typical starch digestion curves over the 360 min digestion are shown in Figure 3. Initially, digestion is rapid with little difference between the pastas up to ~30 min, followed by a lower rate with the data following an exponential curve. Up to 50 min into the starch digestion, only SBEIIa was showing signs of slowing its rate and this continued reaching a lower final digestion compared to Svevo. The curves for the two Svevo pasta’s overlap, as expected while SSIIa and especially the SBEIIa, show lower rates and extent of digestion. For example, after 250 min <40% of starch was digested for SBEIIa while ~70% is digested in Svevo and ~58% in SSIIa. These plots are typical for pasta and the slow starch digestion in pasta is due to the compact microstructure of pasta and because the starch is embedded in a gluten matrix [32,54].

The transformation of the data into logarithm of slope plots reveals two distinct linear steps where ~20–40% of starch is digested in the first phase which is faster (k_1_) and the remainder of the starch digested in a second stage, with a slower rate constant (k_2_) (Table 5). The areas under the curves (AUC) allow a comparison of the extent of starch digestion in the pasta samples and slower digestion would appear as lower AUC values. The AUC values reflect these changes and can be normalised to the AUC in Svevo 2016. For the two Svevo samples, the k_1_ and k_2_ values are very similar as are the AUC and shows that despite different growing season, this did not impact on starch digestion significantly. However, both HA samples have significantly lower AUC than Svevo controls with SBEIIa having the lowest AUC by a significant margin (Table 4). Interestingly, k_1_ and k_2_ values for SBEIIa are faster than all samples but the extent of digestion (C∞ %) is much reduced, suggesting that what is available for digestion is more rapidly digested and that not digested is resistant starch. To the best of our knowledge, there are no in vitro studies focused on the digestion of high amylose pasta.

Hoebler et al. [55] showed that bread rich in amylose had a lower starch degradation compared to control bread. Corrado et al. [56] reported that the starch amylolysis rate and extent were lower for SBEIIa/b-AB compared to those of the control. In rice, the starch digestion rate (k) decreased in flour blend with an increased amount of the HA maize starch [57]. The reason why starch digestibility is reduced with higher amylose content could be due to the strong interaction among the linear polymers of amylose (retrogradation) and between amylose and lipids that results in complex formation on the surface of starch granules [58].

### 3.3. Impact of Elevated Amylose on Pasta In Vivo Starch Digestion and Glycaemic Index

The best measure of a foods glycaemic index is to perform a test with human subjects. The GI test was developed to rank equal carbohydrate portions of different foods according to the extent to which they increase blood glucose levels after the test food is ingested and glucose moves from the intestines into the cardiovascular system. High GI foods are rapidly digested and this produces a sudden and large spike in blood glucose followed by a sharp fall, often achieving a lower blood glucose than basal (pre-ingestion) glucose. In contrast, a low GI food is more slowly digested and this results in a more gradual and lower elevation of blood glucose. Dieticians are using food GI values in planning diets suitable for people with diabetes. Long term epidemiological studies have shown that consumption of high GI impact foods which causes surges in blood glucose and insulin levels, increases the risk of developing type II diabetes, heart disease, obesity, and certain cancers [59,60]. Therefore, the development of low-GI foods could assist with the prevention and treatment of these diseases. In our study, we prepared the semolina and pasta under the same processing conditions, with only water and semolina in the pasta mix, since these factors can impact a foods GI, so only compositional differences matter, which in this case is the amount of amylose as the glutenin composition is identical amongst the genotypes having a glutenin Glu-A1 null, Glu-B1 7 + 8, and LMW-2 (data not shown).

**Figure 4:**
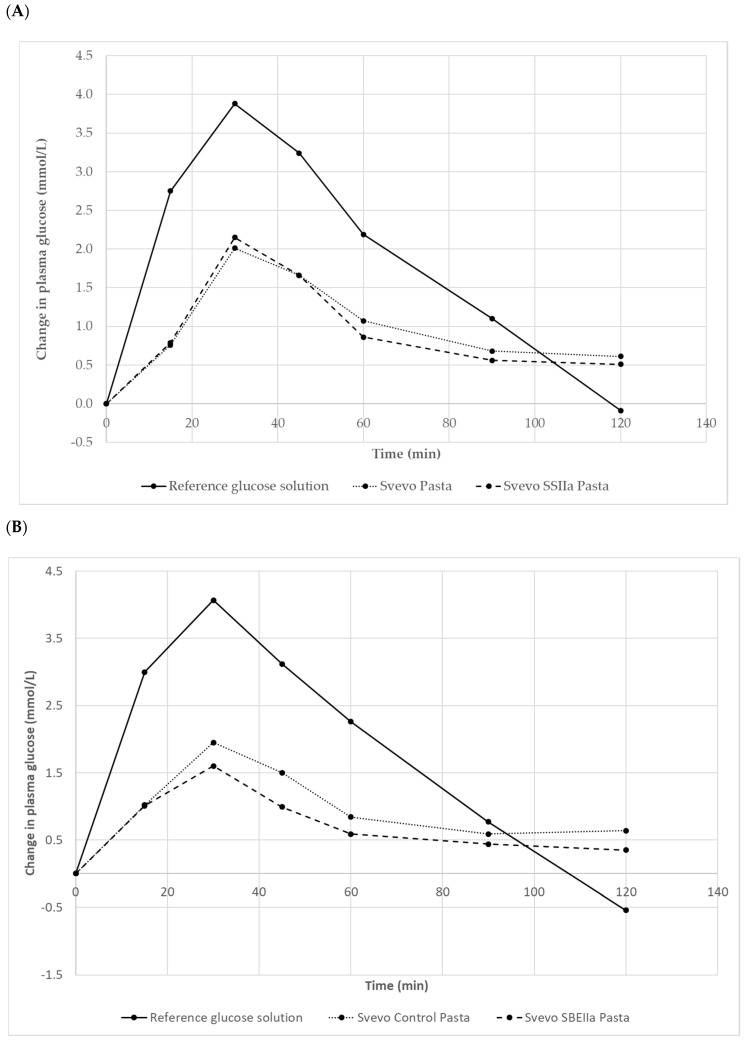
The average plasma glucose response curves (n = 10 subjects) for the equal-carbohydrate portions of the reference food and the two pasta products. (**A**): Svevo 2016 and Svevo SSIIa; (**B**): Svevo 2016 and Svevo SBEIIa, shown as the change in plasma glucose from the fasting baseline level.

Test meal characteristics are reported in Table 6 with a larger portion size of the HA pasta needed to achieve equivalent available starch content. Our in vivo GI testing results are depicted in Figure 4 and Table 7. The reference food (glucose) produced a rapid rise in plasma glucose to a high peak concentration at 30 min then declined reaching below pre-fasting levels after 110–118 min. For the pastas, the glycaemic responses showed a steady rise and much lower peak maxima accompanied by a more gentle return to almost basal glucose levels by 120 min. Svevo 2016 and SSIIa pasta showed similar behaviour in their plasma glucose responses with SSIIa showing slightly lower glucose values between 50–120 min (Figure 4A). Comparing Svevo 2017 with SBEIIa pasta, again we observed similar responses in plasma glucose, but this time, the rise in the SBEIIa pasta was lower and glucose was at a lower level than Svevo 2017 (control) from 15 to 120 min (Figure 4B). Calculation of the mean (10 subjects) GI values for the samples is shown in Table 7. The reference food’s GI value (100) was significantly greater than the average GI for both pasta products (*p* < 0.001). Comparing Svevo 2016 with SSIIa, there was no significant difference in GI (*p* < 0.05) despite showing a lower GI for the latter and being significantly lower in the in vitro assay (Table 5). However, comparing Svevo 2017 with SBEIIa, there was a significantly lower GI in the latter by 10 units. There was no difference in GI between the two Svevo pasta samples, showing that seasonal conditions had no real impact on pasta.

GI (*p* = 0.015) as was noted for the in vitro data. This is the first report of pasta made from 100% durum wheat with a reduction in the GI due to genetic difference in the amylose content. Previous studies have indicated that elevated amylose contents are associated with higher levels of resistant starch in wheat based products (bread and rusks) that induce lower postprandial glycaemic responses [61]. Foods with a GI < 55 are considered to be low-GI foods, which includes all the four pasta samples but the SBEIIa has an even lower GI which is a good achievement.

There were significant correlations between amylose content, resistant starch, in vitro AUC with each other and importantly, with in vivo GI (r 0.90–0.93, *p* < 0.05) but sample size is understandably low due to the high cost of GI testing. Nevertheless, we feel these correlations would prevail even if sample size increased markedly. The higher the amylose, the more resistant starch and the lower the in vitro AUC and extent of digestion. The lower the AUC determined in the in vitro assay the lower the GI with a good prediction (r 0.93). This correspondence between the in vitro and in vivo methods has been shown elsewhere [62]. For more practical purposes such as food consumption, if the amount of these pastas consumed exceeds 50 g, it is better to determine the glycaemic load (GL) value for any sized carbohydrate-containing food. GL = [(GI × amount of available carbohydrate in the portion (50 g) of pasta)/100] and the lower the better, these are shown in Table 7. Dietary intervention studies show that the digestibility of high amylose starch is generally lower than normal starch [28]. As such, foods containing high amylose starch, like the two pastas described here, should help reduce the risk of developing type 2 diabetes. The development and commercialisation of high amylose wheat-based products will allow new products with higher fibre and better sensory acceptability than current wholemeal and wholegrain wheat products designed to increase fibre. Recently, Corrado [56] reported a semolina pudding made from a SBEIIa durum wheat mutant with resistant starch content of ~5%, digested more slowly and to a lesser extent by in vitro test than wild type control. However, they found no difference in the foods GI. Pasta is a low GI traditional Italian food with a crucial role in Mediterranean diet. Pasta consumption has been associated to beneficial effects for human health compared with higher-GI dietary foods. Recently, in a study [63], it has been reported that pasta consumption, in the context of low glycemic index dietary patterns, has no negative effect on body weight and mass. Here, the in vitro and in vivo studies demonstrated that the nutritional value of the pasta was significantly improved with a further reduction of the glycemic index in one of the HA pastas. As the consumption of pasta is constantly growing not only in developed but developing countries, the HA durum wheat genotypes open interesting perspectives in the use of HA pasta products as a vehicle for the prevention of serious non-transmissible diseases, such as colon cancer, type 2 diabetes, and cardiovascular disorders.

## 4. Conclusions

This is the first study of the glycaemic response of pasta prepared from SBEIIa and SSIIa durum wheat mutants with elevated amylose and resistant starch content in a processed pasta food matrix. While the SSIIa mutant increased amylose content, it did not significantly lower spaghetti GI while the SBEIIa mutant, with higher amylose and resistant starch content in the spaghetti, did lower GI. This suggests that a minimum amylose or resistant starch content is needed before GI is lowered in the spaghetti food matrix. We suggest a minimum RS content in spaghetti of ~7% which corresponded to an amylose content of ~58% is needed to lower GI. Both the HA pastas provide higher dietary fibre and resistant starch compared to Svevo and have minimal impacts on pasta technological properties. While the colour of the HA pastas were inferior to Svevo, they were much closer to 100% durum semolina pasta than a typical commercial wholemeal pasta. However, detailed sensory analysis is needed to determine consumer acceptability of this spaghetti. Further evaluation across more environments could be useful. It might then be necessary to further develop the mutants by crossing to improve some of the minor quality deficiencies, but they would have to be re-evaluated for GI and other benefits in future studies, e.g., satiety and long term glyacemic benefits. Compared to typical commercial wholemeal pasta, these HA pastas would be much more visually appealing while providing additional nutritional benefits.

## Figures and Tables

**Table 1:** Semolina properties of HA and control genotypes.

Sample	SP	Amylose (%)	Protein (11%mb) ^a^	Ash (14%mb)	FWA (14%mb)	TDF (%)	TIF (%)	TSF (%)	Granularity (g/100 g)					
									500 µm	425 µm	315 µm	250 µm	180 µm	<180 µm
Svevo2016	8.93 ± 0.61 ^a^	34.0 ± 1. 7 ^a^	12.9	0.70 ± 0.049	59.4 ± 0.14 ^a^	nd	nd	nd	0.07	4.1	33.8	27.5	18.6	15.3
Svevo SSIIa	6.62 ± 0.69 ^b^	43.5 ± 1.5 ^b^	14.2	0.82 ± 0.014	74.3 ± 0.28 ^b^	nd	nd	nd	0.01	2.0	35.3	39.1	17.8	5.4
Svevo2017	10.56 ± 0.59 ^a^	33.3 ± 1.3 ^c^	12.8	0.53 ± 0.012	57.0 ± 0.14 ^c^	2.5 ^a^	1.4 ^a^	1.1 ^a^	0.01	0.06	43.2	36.1	17.4	2.7
Svevo SBEIIa	5.14 ± 0.45 ^b^	57.8 ± 1.5 ^d^	15.4	0.81 ± 0.042	77.2 ± 0.21 ^d^	5.7 ^b^	3.8 ^b^	1.9 ^b^	0.02	0.02	44.3	34.1	17.9	3.3

SP = swelling power; FWA = Flour Water Absorption; TDF = total dietary fibre; TIF = total insoluble fibre; TSF = total soluble fibre. Numbers in the same column with different superscript letters indicate significant differences (*p* < 0.05). ^a^ Measurement was done in commercial lab and precision of measurement for protein is with cv 1.3%. nd = not determined.

**Table 2:** Pasta properties of HA and control genotypes.

Sample	Field Season	Dry Pasta				
		DP-L *	DP-a *	DP-b *	RS% (dm)	TS% (dm)
Svevo	2016	70.12 ± 0.30 ^a^	0.29 ± 0.14 ^a^	44.59 ± 0.62 ^a^	0.73 ± 0.01 ^a^	73.4 ± 0.06 ^a^
Svevo SSIIa	2016	67.01 ± 0.68 ^b^	2.13 ± 0.66 ^b^	38.31 ± 1.05 ^b^	2.06 ± 0.01 ^b^	67.3 ± 1.42 ^b^
Svevo	2017	71.57 ± 0.17 ^c^	−1.95 ± 0.05 ^c^	49.06 ± 0.45 ^c^	0.21 ± 0.02 ^c^	75.6 ± 0.78 ^a^
Svevo SBEIIa	2017	64.49 ± 0.92 ^d^	1.53 ± 0.27 ^d,b^	39.99 ± 1.36 ^d^	7.36 ± 0.10 ^d^	66.4 ± 0.50 ^b^
Commercial wholemeal		44.66	15.25	18.66	nd	nd

DP-L * = dry pasta lightness; DP-a * = dry pasta redness; DP-b * = dry pasta yellowness; RS% = percentage resistant starch; TS = total starch. Numbers in the same column with different superscript letters indicate significant differences (*p* < 0.05).

**Table 3:** Pasta cooking properties of HA and control genotypes.

Pasta	Field Season	FCT (s)	Firmness			Overcook Tolerance	Stickiness		Cooking Loss (%)	Water Absorption
			PH (g)	Area (g/s)	PH/P		PH (g)	Area (g/s)		
Svevo	2016	661 ± 50	1334 ± 65 ^a^	615 ± 22 ^a^	103 ± 5.1 ^a^	52 ^a^	17.0 ± 1.8 ^a,b^	9.3 ± 0.68 ^a^	4.6 ± 0.11 ^a^	146 ± 2.96 ^a^
Svevo SSIIa	2016	578 ± 20	1139 ± 82 ^b^	566 ± 51 ^b^	80 ± 5.8 ^b^	52 ^a^	14.7 ± 1.5 ^a^	5.4 ± 0.83 ^b^	6.9 ± 0.01 ^b^	123 ± 0.01 ^b^
Svevo	2017	761 ± 19	1261 ± 37 ^c^	539 ± 16 ^c^	99 ± 2.9 ^c^	54 ^b^	18.7 ± 1.5 ^b^	6.8 ± 2.2 ^c,b^	4.8 ± 0.30 ^a^	158 ± 0.38 ^c^
Svevo SBEIIa	2017	678 ± 40	1124 ± 82 ^b^	544 ± 51 ^c^	73 ± 5.3 ^d^	49 ^c^	15.9 ± 1.3 ^a^	5.7 ± 1.0 ^c,b^	6.6 ± 0.14 ^b^	120 ± 1.53 ^b^

Data are mean ± stdev. FCT = Fully cooked time; PH = peak height; PH/P = PH divided by semolina protein. Numbers in the same column with different superscript letters indicate significant differences (*p* < 0.05).

**Table 4:** Starch gelatinisation properties of genotypes.

	T_onset_ (°C)	T_peak_ (°C)	T_end_ (°C)	Enthalpy (J/kg)
Svevo 2017	52.76 ± 0.46 ^b,c^	59.51 ± 0.47 ^a,b^	66.78 ± 0.05 ^a^	11.71 ± 0.38 ^b,c^
Svevo SBEIIa	52.27 ± 0.19 ^b^	65.49 ± 0.23 ^d^	88.83 ± 0.23 ^d^	7.92 ± 1.40 ^a,b^
Svevo 2016	53.66 ± 0.56 ^c^	59.91 ± 0.19 ^b,c^	67.81 ± 0.53 ^a,b^	10.91 ± 1.42 ^a,b,c^
Svevo SSIIa	47.04 ± 0.08 ^a^	60.93 ± 0.40 ^c^	69.57 ± 0.18 ^c^	7.23 ± 1.50 ^a^

Numbers in the same column with different superscript letters indicate significant differences (*p* < 0.05).

**Table 5:** Kinetic parameters of digestibility of different pasta products.

Pasta	Total Area under Digestion Curve	Normalised Area	C∞ % I	k_1_	C∞ % II	k_2_
Svevo 2016	19,851 ^a^	1.00	38.9	0.02756	83.2	0.00654
Svevo SSIIa	18,652 ^b^	0.94	35.4	0.02554	81.0	0.00574
Svevo 2017	20,572 ^a^	1.04 (1.00)	37.2	0.02670	87.1	0.00615
Svevo SBEIIa	12231 ^c^	0.62 (0.59)	24.3	0.03470	45.2	0.00831

Numbers in the same column with different superscript letters indicate significant differences (*p* < 0.05). k_1_ and k_2_ refer to starch digestion rate constants at each phase; C∞ % I and C∞ % II refer to estimated % of starch digested at each phase.

**Table 6:** The weight and carbohydrate contents of the test portions of the reference food (glucose) and the HA and control pasta products, calculated using manufacture’s data.

Test Food	Available Carbohydrates for 100 Grams (g)	Portion Size (g)	Available Carbohydrates in Test Portion (g)
Glucose (ref.)	97.30	51.4 g glucose 250 mL water	50.0
Svevo 2016	64.19	77.9 g dry pasta	50.0
Svevo SSIIa	57.35	87.2 g dry pasta	50.0
Svevo 2017	74.05	67.5 g dry pasta	50.0
Svevo SBEIIa	65.27	76.6 g dry pasta	50.0

**Table 7:** Glycaemic index (GI) and glycaemic load (GL) of the reference food and the two pasta samples.

Pasta Sample (Amylose%)	GI	GL
Svevo 2016 (33)	52 ± 3 ^a^	17
Svevo SSIIa (44)	49 ± 3 ^a^	14
Glucose	100 ± 0 ^b^	
Svevo 2017 (32)	48 ± 4 ^a^	18
Svevo SBEIIa (58)	38 ± 3 ^c^	12

Data are mean ± SE. Values with alike superscript letters in the same column are not different, *p* < 0.05.

## Supplementary Materials

The following are available online at https://www.mdpi.com/2304-8158/9/6/693/s1, Table S1: Decadal values of temperature and precipitation recorded in the experimental field from November 2015/2016 to June 2016/2017.
