# Supplementary material for: Can Manipulation of Durum Wheat Amylose Content Reduce the Glycaemic Index of Spaghetti?
Source: Foods. 2020 May 28;9(6):693. doi: 10.3390/foods9060693 (PMC7353610; doi:10.3390/foods9060693)
Supplement: Supplementary file 1 [file foods-09-00693-s001.pdf]

Table S1. Decadal values of temperature and precipitation recorded in the experimental field from November 2015/2016 to June 2016/2017.

Month	Decade	Rain (mm)	Temp. Min (°C)	Temp. Max (°C)
**2015-2016**				
November	1	0.2	9.1	20.9
November	2	0.6	6.6	17.8
November	3	23.4	3.4	11.7
December	1	1.2	4.1	13.8
December	2	0.6	2.6	14.1
December	3	1.6	1.6	13.5
January	1	19.4	5.5	12.4
January	2	27.4	-0.5	9.7
January	3	0.4	2.6	12.5
February	1	15.0	3.3	14.1
February	2	43.4	5.5	13.9
February	3	82.8	4.9	15.1
March	1	27.4	2.8	12.9
March	2	2.0	4.4	15.2
March	3	0.4	5.4	17.7
April	1	0.0	8.4	22.7
April	2	1.4	7.5	21.9
April	3	15.0	7.8	18.6
May	1	27.6	9.8	21.3
May	2	38.0	9.7	20.3
May	3	0.8	10.8	24.4
June	1	45.8	12.6	24.1
June	2	0.4	13.3	25.8
June	3	8.4	17.6	31.8
**2016-2017**				
November	1	5.0	9.0	17.5
November	2	44.4	5.6	14.6
November	3	19.4	5.9	14.9
December	1	0.0	3.5	13.7
December	2	5.2	3.5	11.8
December	3	0.4	1.3	13.1
January	1	6.8	-2.5	6.9
January	2	18.2	-0.4	7.9
January	3	0.2	1.9	12.1
February	1	38.6	7.5	12.6
February	2	4.0	2.2	14.5
February	3	1.4	3.8	14.8
March	1	41.2	4.1	15.6
March	2	0.0	4.1	17.2
March	3	0.0	5.4	19.9
April	1	3.6	6.9	20.4

April	2	2.4	6.1	19.9
April	3	22.6	4.8	18.4
May	1	17.4	8.3	21.5
May	2	0.0	11.9	25.4
May	3	1.0	12.0	27.0
June	1	0.0	13.2	28.7
June	2	0.0	15.7	31.6
June	3	15.6	17.0	31.3
